# Salvage Third-Patch Repair for Left Ventricular Free Wall Rupture After Double-Patch Septal Reconstruction

**DOI:** 10.1016/j.atssr.2025.11.029

**Published:** 2025-12-20

**Authors:** Shun Iwai, Shin Yajima, Daisuke Yoshioka, Takuji Kawamura, Ai Kawamura, Yusuke Misumi, Shunsuke Saito, Shigeru Miyagawa

**Affiliations:** Department of Cardiovascular Surgery, University of Osaka, Suita, Japan

## Abstract

Ventricular septal perforation (VSP) is a rare but fatal complication of acute myocardial infarction. An 83-year-old man experienced VSP with septal dissection after an anteroseptal infarction. Two weeks later, a double-patch repair using the sandwich technique was performed. Intraoperative residual leakage into the dissected septum caused left ventricular rupture, which was successfully repaired by a third bovine pericardial patch through a left ventricular incision for closure. Postoperative echocardiography confirmed no residual shunt and preserved ventricular function. This case underscores the challenges of VSP with septal dissection and emphasizes the value of an additional patch to prevent leakage and rupture.

Ventricular septal perforation (VSP) is a rare complication occurring in approximately 0.17% to 0.27% of myocardial infarctions.^1^ Despite advances in surgical techniques and perioperative management, postoperative mortality rate remains high, exceeding 40%.^1^ Double-patch repair using the right ventricular (RV) approach has demonstrated favorable outcomes in selected cases.^2^ Here we report a case of left ventricular (LV) free wall rupture after double-patch repair for VSP with intraseptal dissection that was successfully managed by an additional patch through the LV approach.

An 83-year-old man with severe chronic obstructive pulmonary disease and diabetes mellitus underwent cholecystectomy for calculous cholangitis at a referral hospital 1 month before transfer. His postoperative course was complicated by retrograde cholangitis. Intravenous antibiotics were administered, and endoscopic nasobiliary drainage was performed. Two days before transfer, he experienced sudden hypotension. Chest radiography revealed pulmonary congestion. Transthoracic echocardiography demonstrated an anteroseptal infarction with VSP and interventricular septal (IVS) dissection (Figures 1A, 1B). The patient was transferred to our institution (Department of Cardiovascular Surgery, University of Osaka, Suita, Japan) for definitive management of postinfarct VSP. Contrast-enhanced computed tomography confirmed IVS dissection, with the tract extending from the upper left to the lower right IVS (Figure 1C). The pulmonary-to-systemic flow ratio was 2.0. Coronary angiography revealed total occlusion of the left anterior descending (LAD) artery septal branch, a finding consistent with myocardial infarction.

**Figure 1:**
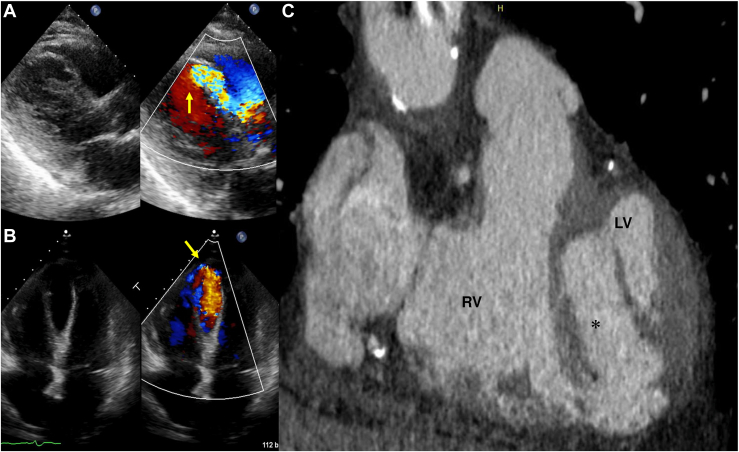
Transthoracic echocardiography demonstrating (A) a shunt (arrow) from the left ventricle (LV) to the dissected interventricular septum and (B) a shunt (arrow) from the dissected septum into the right ventricle (RV). (C) Contrast-enhanced computed tomography showing the communication between the LV and RV through the dissected interventricular septum (asterisk).

On admission, the patient experienced hemodynamic collapse, necessitating extracorporeal membrane oxygenation (ECMO) and Impella 5.5 device support (Abiomed). ECMO was successfully discontinued after 3 days. To allow myocardial stabilization and scar formation, surgical VSP repair was scheduled 2 weeks after infarction under Impella-assisted support.

The Impella device was removed through a median sternotomy and using cardiopulmonary bypass. The VSP was approached through the RV free wall after cardioplegic arrest. A right-sided VSP, approximately 1 cm in diameter, was identified at the apex. The right IVS incision was extended cranially by 3 cm to expose the left VSP. The dissected IVS exhibited a “3-cavity” dissection.

Repair was performed using the sandwich patch technique, with a bovine pericardial patch secured to the LV side of the IVS and the anterior LV wall, and a second patch was placed on the RV side. The intervening dissected cavity was filled with BioGlue (CryoLife). Sutures were passed through both patches.

After unclamping, spontaneous cardiac activity resumed. However, pulsatile bleeding was observed at the RV base medial to the LAD artery. Transesophageal echocardiography revealed residual blood flow within the dissected cavity, indicating free wall rupture. The right ventricle was reopened, and although no bleeding was initially observed in the right patch, removal of BioGlue from the second patch revealed pulsatile blood flow, indicating LV communication. After reinduction of cardiac arrest, the left ventricle was incised between the LAD artery and the first diagonal branch. Although no patch detachment or residual perforation was evident, an endothelial defect was identified on the superior margin of the first patch, presumed to be the leakage source. A third bovine pericardial patch was placed to cover the endocardial defects. The LV incision was closed with felt strip–reinforced mattress sutures, and the aortic cross-clamp was released. On reperfusion, no residual flow was observed in the IVS. A second patch and RV closure were performed. A schematic representation of the procedure is shown in Figure 2, and the Video shows the operative procedure. Transesophageal echocardiography confirmed cessation of intracavitary flow. The patient was transferred to the intensive care unit while undergoing ECMO and intraaortic balloon pump support for hemodynamic stabilization and LV unloading. ECMO and intraaortic balloon pump support were successfully discontinued on postoperative days 4 and 9, respectively.

**Figure 2:**
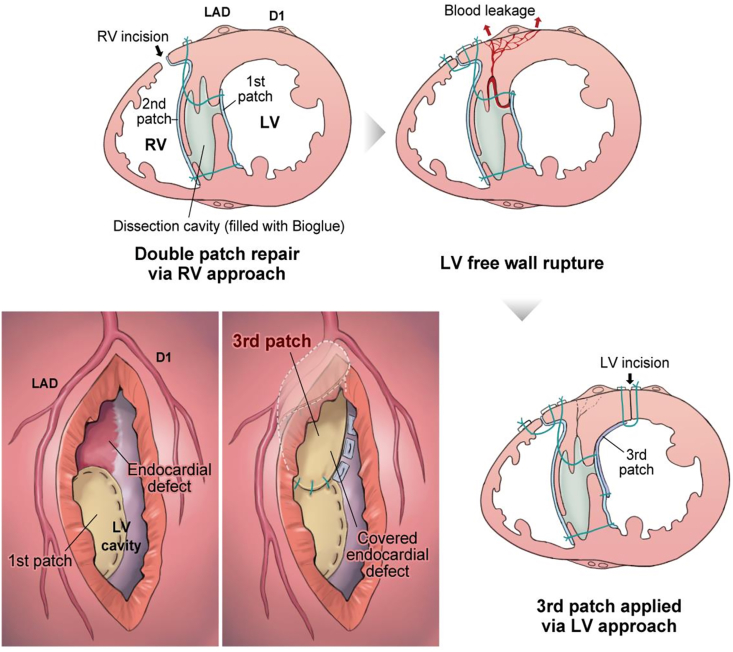
Double-patch ventricular septal perforation repair through a right ventricular (RV) incision. A bovine pericardial patch was placed on the left ventricular (LV) side and another on the RV side of the interventricular septum. After aortic cross-clamp release, pulsatile bleeding was observed at the RV base medial to the left anterior descending (LAD) artery. An endothelial defect was found at the superior aspect of the first patch. A third bovine pericardial patch was applied to cover the endothelial defect area. (D1, first diagonal branch.)

Postoperative transthoracic echocardiography demonstrated an absence of residual shunt and preserved LV function (Figure 3). Despite his initial recovery, the patient died of sepsis secondary to persistent refractory cholangitis 68 days postoperatively.

**Figure 3:**
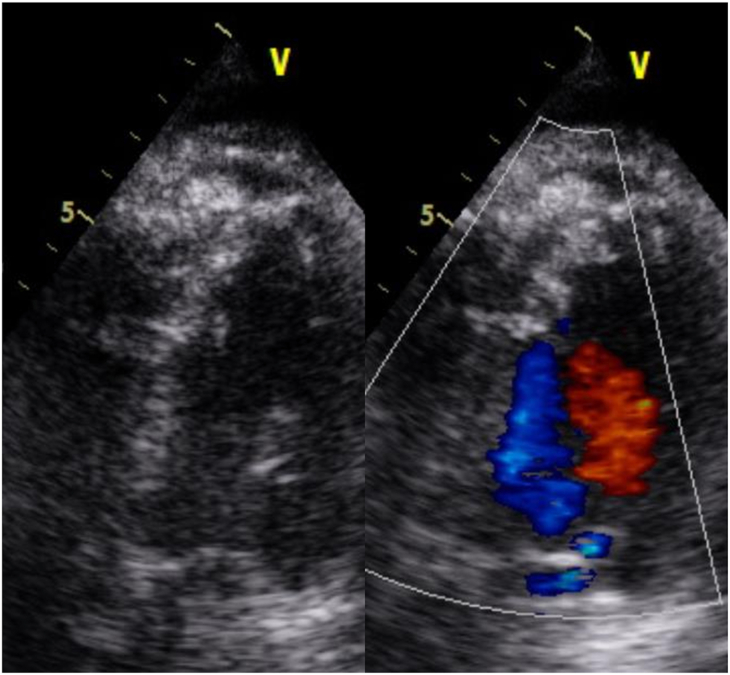
Postoperative transthoracic echocardiography showing the repaired dissected interventricular septum with no residual shunt. (5, 5 cm; V, ventral side).

## Comment

Repairing VSP with IVS dissection presents technical difficulty, with residual shunting remaining a major postoperative concern. Hosoba and colleagues^2^ reported favorable outcomes for simple VSP by using double-patch repair through an RV incision, and they achieved complete closure with a 20% 30-day mortality rate. Conversely, Yamaguchi and colleagues^3^ described a LV pseudofalse aneurysm developing 3 months after extended sandwich patch repair, likely the result of incomplete LV entry closure. Yamaguchi and colleagues^3^ emphasized meticulous LV inflow tract closure, adequate BioGlue application, and the use of sufficiently large patches during RV repair to minimize leakage. In complex cases involving dissected IVS, intraoperative LV rupture may occur secondary to residual leakage.

In our case, repair was intentionally delayed by 2 weeks to allow myocardial healing, thus potentially contributing to incomplete entry closure. Arnaoutakis and colleagues^4^ reported a 54.1% mortality rate for surgery within 1 week of infarction vs 18.4% when surgical treatment was delayed. Histopathologically, myofibroblast differentiation and extracellular matrix deposition, within 7 to 10 days, reinforce the necrotic myocardium,^5^ thus supporting the rationale for delayed intervention.

Unlike Yamaguchi and colleagues,^3^ we used an LV incision that permitted direct visualization of endocardial defects and secure patch placement. Although double-patch repair is typically effective for simple VSP,^2^ residual shunting, aneurysm formation, or rupture may occur in cases complicated by IVS dissection. In such settings, a larger patch or an LV approach may provide more definitive closure and reduce the risk of postoperative complications.
